# Covariation in Plant Functional Traits and Soil Fertility within Two Species-Rich Forests

**DOI:** 10.1371/journal.pone.0034767

**Published:** 2012-04-03

**Authors:** Xiaojuan Liu, Nathan G. Swenson, S. Joseph Wright, Liwen Zhang, Kai Song, Yanjun Du, Jinlong Zhang, Xiangcheng Mi, Haibao Ren, Keping Ma

**Affiliations:** 1 State Key Laboratory of Vegetation and Environmental Change, Institute of Botany, The Chinese Academy of Sciences, Xiangshan, Beijing, China; 2 Department of Plant Biology, Michigan State University, East Lansing, Michigan, United States of America; 3 Smithsonian Tropical Research Institute, Balboa, Panama; 4 Yantai Institute of Coastal Zone Research, The Chinese Academy of Sciences, Yantai, Shandong, China; 5 Graduate University of The Chinese Academy of Sciences, Beijing, China; USDA-ARS, United States of America

## Abstract

The distribution of plant species along environmental gradients is expected to be predictable based on organismal function. Plant functional trait research has shown that trait values generally vary predictably along broad-scale climatic and soil gradients. This work has also demonstrated that at any one point along these gradients there is a large amount of interspecific trait variation. The present research proposes that this variation may be explained by the local-scale sorting of traits along soil fertility and acidity axes. Specifically, we predicted that trait values associated with high resource acquisition and growth rates would be found on soils that are more fertile and less acidic. We tested the expected relationships at the species-level and quadrat-level (20×20 m) using two large forest plots in Panama and China that contain over 450 species combined. Predicted relationships between leaf area and wood density and soil fertility were supported in some instances, but the majority of the predicted relationships were rejected. Alternative resource axes, such as light gradients, therefore likely play a larger role in determining the interspecific variability in plant functional traits in the two forests studied.

## Introduction

The distribution of species and communities along environmental gradients is a central focus in ecology. The distribution of species is expected to be determined by the distribution of resources. The functional strategy of a species will dictate its resource use and therefore its location along a resource axis or resource axes. Thus function should vary predictably along these gradients. This has lead to a tradition in plant ecology of predicting and analyzing the geographic distribution of functional strategies [1], [2].

The relationship between plant function traits and environmental gradients has been quantified for a number of plant traits using large-scale datasets. Evidence from these broad-scale functional trait analyses suggest that the mean functional trait value of an assemblage changes predictably along environmental gradients. For example, leaf and wood traits, seed mass and maximum height have been shown to vary predictably with mean annual temperature [3]–[10]. Additional studies have also examined the relationship between leaf and wood traits with soil nutrient levels. Leaf economic traits related to resource acquisition such as specific leaf area, leaf nitrogen content and leaf phosphorus are positively correlated with soil nutrient content and these relationships were stronger than those with climatic gradients [11]. Wood density, which is negatively correlated with volumetric growth rates, is negatively correlated with nitrogen and phosphorus levels across the Amazon Basin [12]. A running theme in many of these papers is there is a trade-off between the structural allocation and demographic rates based on the resource availability. Specifically, species that favor high resource environments should have higher growth and mortality rates where biomass is allocated to producing a large amount of small seeds that germinate quickly, structurally cheap leaves that have high specific leaf areas but photosynthesize at a high rate, and structurally cheap wood that permits rapid volumetric growth into the canopy. In contrast, species that favor low resource environments should be characterized by ecological strategies that increase structural investment at the cost of decreased resource acquisition and demographic rates. While many of the above studies have supported the expected relationships between environmental gradients and plant traits across broad gradients, this work has also demonstrated that a tremendous level of inter-specific variation occurs within locations along the gradient [13].

The large inter-specific trait variation within sites in global datasets could be the result of trait – environmental gradient relationships on local scales and how different ecological strategies related to resource acquisition and demographic rates sort out along important resource axes. For example, given the previous research showing strong and consistent relationships between plant traits and soil nutrients on global scales, it is expected that local scale plant trait distributions should also vary predictably along local scale soil nutrient gradients. In particular, we predict that individual species with plant traits associated with high rates of resource acquisition and growth such as high values of specific leaf area, maximum height and leaf area and low values of seed mass and wood density are predicted to occur on soils with high nutrient content. Conversely, species with low values of specific leaf area, maximum height and leaf area and high values of seed mass and wood density are expected to be located in soils with low nutrient levels.

Here, we integrate tree distribution and soil nutrient data with five plant functional traits – specific leaf area, maximum height, leaf area, seed mass and wood density to test the predicted relationships among local-scale gradients in soil nutrient levels. In particular, we quantify: (1) the correlation between species mean trait values and their mean position on soil nutrient gradients and (2) the correlation between the mean trait value in 20×20 m quadrats and the soil nutrient level in that quadrat. The analyses are performed separately in two forest inventory plots. The two forest plots were chosen for two important reasons. First, they share similar forest inventory, trait collection and soil nutrient mapping protocols making a comparative study feasible. Second, the forests are vastly different in their topographic heterogeneity thereby allowing us to determine whether the degree of local habitat heterogeneity influences the strength of trait-soil relationships. We first test the above predictions using species-level data and then ask whether the species-level relationships scale-up to the quadrat-level where the mean trait value within a quadrat can be predicted based on the soil nutrient levels in that quadrat.

## Materials and Methods

### Research Sites

**Figure 1:**
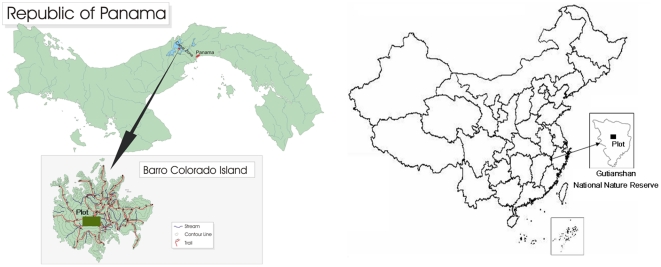
A map of the geographic location of the 50-ha BCI plot, Barro Colorado Island, Panama and the 24-ha GTS plot, Gutianshan National Nature Reserve, China.

The datasets used in this study were compiled from two permanent large forest dynamics plots in tropical and subtropical forests. The Barro Colorado Island (BCI) 50-ha forest dynamics plot (9°10′N, 79°51′W) is located on well-weathered kaolinitic Oxisols on Barro Colorado Island, Panama (Fig. 1), and it is characterized as a lowland semideciduous moist forest. In the 1000×500 m rectangular area, the plot spans an altitudinal range of 120 to 160 m and the slope ranges from 7° to 20°. Daily maximum and minimum temperatures average 30.8°C and 23.4°C, respectively. Annual rainfall averages 2600 mm, with just 10% of the annual total falling during a 4-mo dry season. The BCI plot was first censused in 1981/82 [14]. All trees with a diameter at breast height (dbh) ≥1 cm were measured, identified and mapped. A second census was performed in 1985 and censuses have been repeated every 5 years thereafter. Here we use the 2005 census, which includes 208,387 individual trees belonging to 299 species.

The Gutianshan (GTS) 24-ha permanent plot (29°15′N, 118°07′E) is located in the old-growth forest of Gutianshan National Nature Reserve, Kaihua County, Zhejiang Province, Southeast China (Fig. 1), and it is characterized as a subtropical evergreen broad-leaved forest. The GTS forest plot contains approximately 140,000 individual trees (dbh≥1 cm), representing 49 families, 103 genera and 159 species in the plot. It was established in the summer of 2005, following the same protocol as for BCI [15], [16]. In the 600×400 m rectangular area, the plot spans an altitudinal range of 446.3 to 714.9 m and the slope ranges from 13° to 62°. The mean annual temperature in the Gutianshan Reserve is 15.3°C. The hottest month is July (mean temperature of 27.91°C), and the coldest is January (mean temperature of 4.31°C). Annual precipitation averages 1963.7 mm, with a dry period between October and February. The major soils can be classified into four types: red soil, red-yellow soil, yellow-red soil and marsh soil [15].

### Plant Functional Traits

We measured leaf area (LA), specific leaf area (SLA), wood density (WD), seed mass (SM) and maximum height (H_max_) for species at both sites. The trait collection protocols for BCI are described in Wright et al. [17], and the GTS collection protocols followed Cornelissen et al. [18] with the exception of WD which followed the protocols of Wright et al. [17]. Below we briefly describe the collection methods and sample sizes for the GTS plot.

Leaf area and SLA were measured using at least ten mature leaves collected from the tallest portion of the canopies of 5–10 of the largest individuals of each species. The SLA was calculated as mean of fresh leaf area divided by the leaf dry mass without the petiole. The LA was measured as the mean leaf surface area without petioles for each species. The SM was calculated by collecting 30 to 200 mature, fresh seeds from more than five individual trees of each species in or near the plot. We removed appendages and oven dried seeds for 48 h at 80°C. The SM value is the mean value over all seeds of each species. The WD was calculated by collecting wood samples from 5 to 10 individuals for each species in the area surrounding the plot using methods described in Wright et al. [17]. The H_max_ values for GTS were estimated using values reported in the Flora of China [19] and the Flora Reipublicae Popularis Sinicae [20].

### Phylogenetic Trees

Two phylogenetic trees were utilized in this study. Specifically, we utilized a phylogenetic tree from Kress et al. [21] for the BCI plot. This phylogeny was constructed using a DNA supermatrix composed of three sequence regions - rbcL, matK, and trnH-psbA. The supermatrix and the software RAxML [22] were used to construct a maximum likelihood phylogenetic tree. We constructed a phylogenetic tree for the GTS forest plot species following the same methodology as Kress et al. [21]. Figures of both phylogenetic trees are available in the supplemental material (see Figure S1& Figure S2).

### Soil Fertility

Soil samples were collected and analyzed following the protocols established by the Center for Tropical Forest Science (CTFS) in both plots (http://ctfs.si.edu/datasets/bci/soilmaps/BCIsoil.html) [23]. However, the sampling design and intensity differed. At BCI, the plot was divided into a 50×50 m grid, grid intersections were basal collection points, and additional collection points were marked at 2 m, 8 m or 20 m along a random compass direction from each basal point. Thus, 300 points were sampled in the 50-ha plot. At GTS, the grid was 30×30 m, the additional collection points were 2, 5 and 15 m along a random compass direction from each basal point, and a total of 892 samples were collected inside the 24-ha plot.

John et al. [23] describe the methods used to process BCI soil samples. At GTS, a 300–400 g topsoil sample was taken from 0–10 cm depth and air-dried. The soil was then sieved with a 2 mm mesh screen. The sieved soil was used to extract available cations. 50 g of the 2 mm-filtered soil was filtered again with 0.15 mm mesh screen for analyses of total C, N, and P. Additional samples from a depth of 15 cm were taken using two polyethene pipes with a diameter of 5 cm. One of these samples was used for extracting NH_4_^+^ and NO_3_^−^ (using 2.0 M KCl on 2 g soil) and measuring gravimetric moisture content and pH value (soil: water was 1∶5). The other sample was sealed and left in the original state for 26–31 days in order to measure N mineralization rates. Available cations were extracted using Mehlich-III extractant solution and elemental analysis was done by Atomic Emission-Inductively Coupled Plasma (AE-ICP) spectrometry. We analyzed NH_4_^+^ and NO_3_^−^ with a Continuous Flow Analyzer in the Key Laboratory of Plant-Soil Interactions, China Agriculture University. We used Walkley-Black method to estimate total C and used the Kjeldahl Nitrogen Determination method to measure total N. Total P was measured by UV-Spectrometer in the State Key Laboratory of Vegetation and Environmental Change, Institute of Botany, CAS. Finally, we obtained the values of 13 soil nutrients (Fe, Mn, Zn, Cu, K, P, Ca, Mg, B, Al, N, pH, Nmin) for both plots by ordinary kriging. Additional detailed information regarding soil data collection can be found in John et al. [23] for BCI and Liwen et al. [24] for GTS.

For each forest plot, we used a principal components analysis (PCA) to extract orthogonal axes of soil fertility and acidity from the 13 measured soil nutrients and to reduce information redundancy. We used the significant PCA axes to characterize soil fertility and acidity for all subsequent analyses.

### Statistical Analyses

Our datasets included mean trait values (T) for each species S (T_S_), soil fertility (F) for each 20×20 m quadrat Q (F_Q_) and the number of individuals of each species in each quadrat (N_SQ_). We used these measured values to calculate mean soil fertilities for each species (F_S_) and mean trait values for each quadrat (T_Q_) as follows:
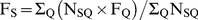
(1)

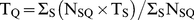
(2)


We first performed a species-level analysis to test our predictions by calculating a Pearson correlation of trait values and mean soil properties for species calculated by weighting the PCA scores of each 20×20 m quadrat by the number of individuals of species in that same quadrat (eqn. 1) The LA and SM values for species in both forest plots were log transformed to satisfy the normality assumption. Next we used phylogenetically independent contrasts (PICs) [25]–[27] to evaluate relationships between measured values of T_S_ and calculated values of F_S_. This second analysis was used to factor out the bias of phylogenetic non-independence and to evaluate the hypothesis that evolutionary changes in trait values were associated with the spatial distribution of species with respect to soil fertility. PIC regressions were forced through the origin [28] and significance was evaluated after removing extreme outliers (absolute value of studentized residual>5) and contrasts with undue leverage (leverage>0.2).

We performed a third correlation analysis to evaluate the relationship between calculated T_Q_ (eqn. 2) and measured/estimated F_Q_. The LA and SM values from the BCI plot were log transformed to satisfy the normality assumption. This quadrat-level analysis was used to test whether quadrat-level trait distributions shift in a predictable direction along the soil fertility and acidity gradients within each forest plot. This analysis was then repeated using torus translation simulations [29]. The procedure included two steps: 1) we moved the true soil map by 20-m increments two-dimensionally, but kept the above trees map still; 2) We recalculated the Pearson correlation between T_Q_ and F_Q_ based on 20×20 m quadrats for each simulation and compared the observed and simulated correlation coefficients. If the rank of the r-value from the true quadrat was higher than 97.5% or lower than 2.5% of the ranks of the simulated r-values (two-tailed test), it was considered that T_Q_ and F_Q_ was significantly correlated. The torus translations maintained the observed spatial distribution of soil fertility and tree distributions, but break their observed dependence by shifting the observed soil fertility distribution on a torus relative to fixed tree distributions.

We performed the three correlation analyses for five plant traits (SM, LA, SLA, WD, H_max_) and the soil properties of the first two principal component axes (see Results: Soil Properties). We therefore used the false discovery rate (FDR) approach to adjust p-values [30], [31]. Except the torus translation simulation, all other reported p-values refer to the adjusted p-values. All analyses were performed in R 2.13.0 (R core team, 2011).

## Results

### Soil Properties

The significant soil PCA axes combined to explain more than 70% the variation of soil nutrients at each site (Table 1). For GTS, the first axis explained 42.2% of the overall variation and represented a general soil fertility axis with negative loadings on most key limiting elements. The second axis explained 17.8% of the overall variation and represented an acidity index with a relatively large negative loading on pH (−0.461) and positive loadings on Fe (0.579) and B (0.610). The third axis explained 12% of the overall variation (Table 1).

**Table 1:** Principal component analyses for 13 soil fertility for the GTS plot and the BCI plot.

	GTS			BCI		
	PC1	PC2	PC3	PC1	PC2	PC3
Al	−0.012	0.172	**−0.396**	0.167	**0.348**	**0.600**
B	−0.050	**0.610**	0.109	**−0.339**	−0.131	0.111
Ca	**−0.391**	−0.031	0.150	**−0.355**	0.049	−0.041
Cu	**−0.321**	0.031	**0.334**	**−0.305**	0.218	0.115
Fe	0.039	**0.579**	0.001	**−0.278**	**0.336**	0.133
K	**−0.348**	0.086	−0.075	**−0.353**	0.009	0.013
Mg	**−0.409**	−0.002	0.064	**−0.331**	0.044	−0.028
Mn	**−0.324**	−0.075	**0.374**	**−0.254**	**0.245**	0.206
Zn	**−0.359**	0.165	−0.096	**−0.338**	−0.006	−0.048
N	**−0.324**	0.027	−0.088	−0.127	**−0.595**	0.243
Nmin	−0.206	−0.083	**−0.519**	**−0.269**	0.102	−0.109
P	−0.234	−0.024	**−0.511**	0.038	−0.228	**0.692**
pH	−0.125	**−0.461**	0.015	−0.240	**−0.470**	−0.005
Eigenvalue	5.483	2.316	1.566	7.187	1.582	1.44
% explained	42.2	17.8	12	55.3	12.2	11.1

Entries are component loadings; eigenvalues and percentage of variation explained for the three significant principal components for each site. Significant loadings are in boldface type.

For BCI, the PC1 axis explained 55.3% of the overall variation. Similar to the GTS analysis, this axis was also generally indicative of a soil fertility index with most elements decreasing. Again similar to GTS, the PC2 axis for BCI explained more than 12% of the overall variation and was an acidity index with low pH (−0.470) and N (−0.595) and high Al (0.348) and Fe (0.336). The PC3 axis explained about 11% of the overall variation and captured a large correlation between Al (0.600) and P (0.692) (Table 1). This is also similar to the third axis at GTS, which had large loadings of the same sign for Al and P (Table 1).

For both plots, the PC1 axis was negatively related to soil fertility as shown by the negative loadings of most essential nutrients. Therefore, a negative correlation between a mean trait value and the soil fertility PC1 axis meant that trait values were larger on more fertile soils. The PC2 axis was positively related to soil acidity as shown by the negative loading of pH on PC2 (or lower pH for larger values of PC2) (Table 1). Therefore, a negative correlation with the soil acidity PC2 axis meant that larger trait values occurred on less acidic soils. Given the similarities between the loadings of nutrients on the first two PCA axes in both plots and because of their interpretability as general fertility and acidity axes, the following will focus on these first two axes.

### Species-level Relationships between Trait Values and Soil Properties

Species functional trait values (*T_S_*) were unrelated to calculated mean species soil properties (*F_S_*) at BCI (Table 2). For GTS, after the false discovery rate (FDR) correction to p-values, LA was negatively related to the soil fertility axis PC1 (r = −0.220; p = 0.008) and LA (r = −0.275; p<0.001), SLA (r = −0.221; p = 0.008) and WD (r = 0.269; p<0.001) were significantly related to the soil acidity axis PC2 (Table 2). We repeated these analyses for individual soil variables (see Table S1& Table S2). In sum, in both plots several significant relationships were uncovered between individual soil variables and all traits except for H_max_ at GTS and SM at BCI.

**Table 2:** Pearson correlation coefficients between five functional traits and the calculated scores of the two significant principal components of soil fertility and acidity for both GTS and BCI at the species-level (Leaf area and Seed mass of both plots are log_10_transformed).

		GTS		BCI	
		PC1	PC2	PC1	PC2
Leaf area	r	**−0.220**	**−0.275**	−0.067	−0.074
	n	157	157	283	283
	p	0.003	0.000	0.131	0.107
	p-adj	0.008	0.000	0.330	0.330
Specific leaf area	r	0.048	**−0.221**	−0.032	−0.028
	n	157	157	284	284
	p	0.275	0.003	0.296	0.319
	p-adj	0.324	0.008	0.399	0.399
Seed mass	r	−0.105	0.065	0.014	−0.086
	n	141	141	171	171
	p	0.108	0.222	0.428	0.132
	p-adj	0.216	0.324	0.476	0.330
Wood density	r	0.047	**0.269**	−0.048	−0.034
	n	157	157	262	262
	p	0.279	0.000	0.220	0.292
	p-adj	0.324	0.000	0.399	0.399
Maximum height	r	−0.044	0.010	−0.000	−0.118
	n	157	157	283	283
	p	0.292	0.451	0.500	0.023
	p-adj	0.324	0.451	0.500	0.230

Significant correlations are in boldface type (p-value<0.05 after the False Discovery Rate adjustment).

### Phylogenetically Independent Species-level Relationships between Trait Values and Soil Properties

A second set of species level analyses were performed using phylogenetically independent contrasts (PICs) to account for the evolutionary non-independence of species. In the GTS plot, five significant relationships were found between trait contrasts and soil contrasts (*F_S_* from eqn. 1) (Table 3). In particular, significant positive relationships were found between SLA and the soil fertility axis PC1 (r = 0.302; p<0.001), SM and the soil acidity axis PC2 (r = 0.197; p = 0.028) and WD and PC2 (r = 0.286; p<0.001). Negative relationships were found between LA and the soil fertility axis PC1 (r = −0.176; p = 0.036) and SLA and the soil acidity axis PC2 (r = −0.245; p = 0.007).

**Table 3:** Phylogenetically independent contrasts (PICs) between five functional traits and the calculated scores of the two significant principal components of soil fertility and acidity for both GTS and BCI.

		GTS		BCI	
		PC1	PC2	PC1	PC2
Leaf area	r	**−0.176**	0.130	0.032	**0.268**
	n	144	145	253	247
	p	0.018	0.057	0.356	<0.001
	p-adj	0.036	0.076	0.445	<0.001
Specific leaf area	r	**0.302**	**−0.245**	0.077	0.084
	n	143	144	254	252
	p	<0.001	0.002	0.101	0.087
	p-adj	<0.001	0.007	0.144	0.144
Seed mass	r	0.055	**0.197**	**0.352**	**0.274**
	n	131	132	145	148
	p	0.263	0.011	<0.001	<0.001
	p-adj	0.263	0.028	<0.001	<0.001
Wood density	r	0.077	**0.286**	**0.167**	**−0.202**
	n	143	145	232	227
	p	0.178	<0.001	0.005	0.001
	p-adj	0.198	<0.001	0.010	0.003
Maximum height	r	0.130	0.130	0.011	0.008
	n	143	142	151	150
	p	0.061	0.061	0.449	0.463
	p-adj	0.076	0.076	0.463	0.463

Significant correlations are in boldface type (p-value<0.05 after the False Discovery Rate adjustment).

In the BCI plot, five significant relationships were also found between trait contrasts and soil contrasts (Table 3). The soil fertility axis PC1 was positively correlated with SM (r = 0.352; p<0.001) and WD (r = 0.167; p = 0.010). The soil acidity axis PC2 was positively correlated with LA (r = 0.268; p<0.001) and SM (r = 0.274; p<0.001) and negatively correlated with WD (r = −0.202; p = 0.003).

Similar to the non-phylogenetic analyses, we repeated all analyses using individual soil nutrients (see Table S3 & Table S4). Similar to the above non-phylogenetic results, several significant relationships were uncovered in both plots between individual soil variables and all traits except for SM at GTS.

### Relationships between Quadrat-Level Mean Trait Values and Soil Properties

**Figure 2:**
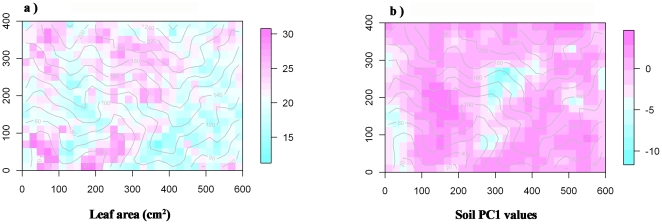
Maps of the quadrat trait and soil fertility patterns. a) The observed leaf area pattern for the GTS plot. b) The soil PC1 values pattern for the GTS plot. The color scale on the right of each map indicates the trait and soil PC1 values. The lines are elevation contour lines at 10-m intervals. See Figure S3 for the complete maps of other traits and the soil PC2 values for GTS plot and Figure S4 for maps of all traits and the soil PC1 and PC2 values for BCI plot.

A second goal of this study was to test whether our predictions regarding species-level trait – soil relationships scale-up to the quadrat-level. Fifteen of the 20 possible relationships were significant after the FDR correction to probability levels (Table 4). In the GTS plot, there was a negative relationship between the soil fertility axis PC1 and LA (Fig. 2; r = −0.394; p<0.001), SM (r = −0.450; p<0.001) and WD (r = −0.179; p<0.001) and positive relationship with SLA (r = 0.224; p<0.001) and H_max_ (r = 0.186; p<0.001). Negative relationships were found between the soil acidity axis PC2 and LA (r = −0.458; p<0.001), SM (r = −0.130; p = 0.001) and H_max_ (r = −0.115; p = 0.003), and a positive relationships with WD (r = 0.325; p<0.001). For the distribution pattern of LA and soil PC1 values see Fig. 2. The spatial distribution of the other traits and soil PC2 values could be found in Figure S3. In the BCI plot, the soil fertility axis PC1 was negatively correlated with H_max_ (r = −0.283; p<0.001) and positively correlated with SLA (r = 0.258; p<0.001) and SM (r = 0.113; p<0.001).The soil acidity axis PC2 axis was positively correlated with LA (r = 0.242; p<0.001) and H_max_ (r = 0.057, p = 0.037), but negatively correlated with WD (r = −0.206; p<0.001). The spatial distribution of all traits and soil PC1 and PC2 values are shown in Figure S4. As with the species-level analyses, all analyses were conducted on individual soil nutrients (see Table S5 & Table S6). At the quadrat-level all traits were correlated with at least one individual soil variable in each forest plot.

**Table 4:** Pearson correlation coefficients between five functional traits and the calculated scores of the two significant principal components of soil fertility and acidity for both GTS and BCI at the quadrat-level (Leaf area and Seed mass of BCI plot are log_10_transformed).

		GTS		BCI	
		PC1	PC2	PC1	PC2
Leaf area	r	**−0.394**	**−0.458**	0.035	**0.242**
	n	598	598	1248	1248
	p	<.001	<.001	0.108	<.001
	p-adj	<.001	<.001	0.135	<.001
Specific leaf area	r	**0.244**	−0.025	**0.258**	−0.001
	n	598	598	1248	1248
	p	<.001	0.271	<.001	0.486
	p-adj	<.001	0.271	<.001	0.486
Seed mass	r	**−0.450**	**−0.130**	**0.113**	0.039
	n	598	598	1248	1248
	p	<.001	0.001	<.001	0.084
	p-adj	<.001	0.001	<.001	0.120
Wood density	r	**−0.179**	**0.325**	0.017	**−0.206**
	n	598	598	1248	1248
	p	<.001	<.001	0.274	<.001
	p-adj	<.001	<.001	0.304	<.001
Maximum height	r	**0.186**	**−0.115**	**−0.283**	**0.057**
	n	598	598	1248	1248
	p	<.001	0.002	<.001	0.022
	p-adj	<.001	0.003	<.001	0.037

Significant correlations are in boldface type (p-value<0.05 after the False Discovery Rate adjustment).

### Torus Translation Simulations

As there is substantial spatial auto-correlation in species distributions and soil nutrient levels, we re-analyzed all of the quadrat-level trait-soil relationships using a torus translation approach. In Table 5, we provide the rank of observed Pearson r-values in the distribution of the randomized r-values for the 10 predictions for both BCI and GTS. The rank value could be used to calculate the significance of the observed correlations. In particular, low ranks or p-values indicated stronger than expected negative correlation and high ranks or p-values indicated a stronger than expected positive correlation. In the GTS plot, the observed significant Pearson correlation r-values between LA and SM and the soil fertility axis PC1 were still significant in the torus simulation (see Table 5; p>0.975 and p = 1.000). The observed relationship between the soil acidity axis PC2 and LA and WD were also significant (see Table 5; p = 1.000 and p<0.025). In the BCI plot, none of the observed r-values were significant after implementing the torus translation simulations and the false discovery rate (FDR) correction to p-values (Table 5).

**Table 5:** Torus translation simulation of the Pearson correlation between traits and the calculated scores of the two significant principal components of soil fertility and acidity shifting at 20 m-scale at the quadrat-level for both GTS and BCI.

		GTS		BCI	
		PC1	PC2	PC1	PC2
Leaf area	r	**599**	**600**	399	163
	n	600	600	1250	1250
	p	0.998	1.000	0.319	0.130
	p-adj	0.993	1.000	0.495	0.325
Specific leaf area	r	31	282	49	485
	n	600	600	1250	1250
	p	0.052	0.47	0.039	0.388
	p-adj	0.081	0.47	0.195	0.495
Seed mass	r	**600**	536	362	509
	n	600	600	1250	1250
	p	1.000	0.893	0.289	0.407
	p-adj	1.000	0.881	0.495	0.495
Wood density	r	576	**2**	561	1176
	n	600	600	1250	1250
	p	0.960	0.003	0.449	0.941
	p-adj	0.920	0.008	0.495	0.803
Maximum height	r	38	561	1218	618
	n	600	600	1250	1250
	p	0.063	0.935	0.975	0.495
	p-adj	0.081	0.919	0.805	0.495

Significant correlations are in boldface type (p-value<0.025 or p-value>0.975 after the False Discover y Rate adjustment).

## Discussion

The distribution of plant species and communities along broad-scale environmental gradients is expected to be determined by the sorting of species along these gradients on the basis of their function (e.g. [4], [8]–[12], [32], [33]). If a similar sorting of species by function occurs on local scales, then this may explain the substantial level of interspecific variation within local sites [13]. An expected mechanism underlying these trends is that species with ‘fast’ leaf, seed and wood economies that have faster resource acquisition and demographic rates should prefer resource rich ends of the gradient and species with ‘slow’ economies that have slower resource acquisition and demographic rates should prefer the resource poor ends of the gradient. The present analysis tested these mechanistic predictions in two forest plots. While the distribution of some plant traits showed significant relationships with local soil gradients, the majority of the expected relationships were not supported. In the following we discuss the results in detail for each forest plot.

### Relationships between Functional Traits and Soil Resource Axes in the Subtropical Gutianshan (GTS) forest plot

Our species-level correlation analyses of the Gutianshan (GTS) forest plot in China supported three of our ten predictions when considering the results of both the species-level analyses and the phylogenetically independent contrasts (PICs) (Table 6). Specifically, leaf area was positively correlated with soil fertility, specific leaf area was negatively correlated with soil acidity (pH) and wood density was positively correlated with soil acidity (pH). Leaf area and specific leaf area (SLA) are known to be correlated with high rates of resource acquisition and growth [11], [17], [34], [35]. For example, species with high SLA values have low structural investment and relatively high photosynthetic and respiration rates, whereas species with low SLA values tend to invest more on leaf structures and have relatively low photosynthetic and respiration rates [2], [18], [36]. It was therefore expected that plants with larger SLA were found in high nutrient soils, whereas the reverse was expected to occur in low nutrient-supply soil. The trade-off between wood density and species growth and mortality rates has been shown in previous studies [37]–[40]. Speices in shaded or arid sites gernerally have smaller vessels and thicker fiber walls, thereby increasing their wood density. In the GTS plot, light wooded species tended to be found on fertile soils suggesting that high resource environments favoured species that allocate less biomass per unit volume and that have higher growth and mortality rates.

**Table 6:** A summary table of whether the predicted correlation results for both the GTS and BCI forest plots were supported in this study.

	Species-level(Table 2)		Species PICs		Quadrat-level		Torus Translation Simulation	
	(Table 2)		(Table 3)		(Table 4)		(Table 5)	
Predicted Correlation	GTS	BCI	GTS	BCI	GTS	BCI	GTS	BCI
Negative LA & PC1	+	NS	+	NS	+	NS	+	NS
Negative LA & PC2	+	NS	NS	−	+	−	+	NS
Negative SLA & PC1	NS	NS	−	NS	−	−	NS	NS
Negative SLA & PC2	+	NS	+	NS	NS	NS	NS	NS
Positive SM & PC1	NS	NS	NS	+	−	+	−	NS
Positive SM & PC2	NS	NS	+	+	−	NS	NS	NS
Positive WD & PC1	NS	NS	NS	+	−	NS	NS	NS
Positive WD & PC2	+	NS	+	−	+	−	+	NS
Negative H_max_ & PC1	NS	NS	NS	NS	−	+	NS	NS
Negative H_max_ & PC2	NS	NS	NS	NS	+	−	NS	NS

The correlations were calculated between the soil PC axes and the species-level or quadrat-level trait value. The table depicts whether the prediction was significant and supported the prediction (+), was significant and did not support the prediction (−) or was non-significant (NS). Phylogenetically independent contrasts (PICs) and torus translation simulations were utilized to correct for evolutionary non-independence in the species-level analyses and spatial auto-correlation in the quadrat-level analyses respectively. LA: leaf area; SLA: specific leaf area; SM: seed mass; WD: wood density; H_max_: maximum height.

### Relationships between Functional Traits and Soil Resource Axes in the Tropical Barro Colorado Island (BCI) forest plot

The species-level correlation analyses of the Barro Colorado Island (BCI) forest plot in Panama found no support for our predictions regarding species traits and soil fertility or acidity in species-level analyses (Table 6), while the PIC analyses provided support for all but three of our predictions. Thus we could only support the predictions that seed mass would be positively related to soil acidity and negatively related to soil fertility and wood density would be negatively related to soil fertility (Table 6). In the low resource environments, large seeds could provide more reserves for individuals early in their life cycle. Small seeds, on the other hand, have the potential advantage of greater dispersal ability and rapid growth in high resource environments [41]–[44].

### Quadrat-Level Trait-Soil Relationships in the Two Forest Plots

A secondary goal of the present study was to determine whether our predictions regarding species-level trait relationships with soil fertility and acidity gradients would scale-up to the quadrat-level. Although we found many significant relationships, the majority of these were non-significant once we accounted for spatial autocorrelation (Table 6). For example, in the GTS forest plot, the relationships between most traits and soil fertility were significant, but after controlling for spatial autocorrelation via a torus translation analysis, only four were still significant and only three of our ten predictions were still supported. This finding was consistent with our findings at species-level. From this, we can infer that for these few trait-soil relationships, it may be that the observed local relationships scale-up to generate the regional scale relationships reported elsewhere.

The quadrat-level results from BCI yielded no support for our predictions once we accounted for spatial autocorrelation. The non-significant co-variation between traits and soil at the quadrat-level may be based on very weak relationships at species-level in the BCI forest plot. A possible explanation for the BCI results is that the location for this forest plot was chosen to be as homogeneous as possible and a large proportion of trees there occur in shaded environments [45]. Therefore, the most important factors influencing the sorting of plant traits may be light levels or other factors rather than soil nutrients. Thus, this level of homogeneity also highlights one weakness of our study. Specifically, while the forest plots being analyzed used standardize tree inventory protocols, they were not set up to standardize the level of environmental heterogeneity. Future comparative research into the relationship between traits and soil nutrient gradients should therefore seek to standardize the level of soil nutrient heterogeneity at the plot-level.

Ultimately, the relationship between traits and soil fertility might be moderated by additional environmental parameters not presently analyzed and likely by the difference in the breadth of various resource axes. This may explain the lack of strong trait – environment relationships in this study. Besides, a potential reason for the different results for the two plots is the difference in terms of seasonality and associated harshness of the abiotic environment.

In summary, plant functional trait research has shown that plant traits vary predictably along broad-scale climatic and soil gradients. The present research predicted that this variation might be explained partly by local-scale soil fertility and acidity gradients. Although we found leaf area and wood density had a consistent and predictable relationship with soil fertility both at species and quadrat-level for GTS, we failed to find support for most predicted relationships between plant traits and soil fertility and acidity axes. In particular, the limited evidence for species-level associations between traits and soil fertility and acidity failed to scale up to the quadrat-level for both the GTS and BCI plots. The general lack of support for the predictions at the BCI forest plot may be due to limited heterogeneity in soil nutrients in this particular forest, but the same cannot be said for the GTS plot as it is quite heterogeneous with rugged terrain. In both plots it is clear that soil nutrients are not the only determinant of plant trait distributions and alternative resource axes, such as light, will have to be considered in future work. Ultimately, while some of our predictions regarding local-scale trait distributions, soil fertility and soil acidity were supported other factors likely play a larger role in determining the large interspecific variation in trait values in the forests studied.

## Supporting Information

Figure S1 **The phylogenetic tree constructed using DNA barcodes of the species in the GTS plot (See details on tree construction in the text).**
(TIF)

Figure S2 **The phylogenetic tree constructed using DNA barcodes of the species in the BCI plot (See details on tree construction in the text).**
(TIF)

Figure S3 **Maps of the quadrat trait and soil fertility patterns for the GTS plot.** Map a), b), c) and d) are the observed SLA, seed mass, wood density and maximum height patterns; and map e) is the soil PC2 values for the GTS plot. The color scale on the right of each map indicates the trait and soil PC2 values. The lines are elevation contour lines at 10-m intervals.
(TIF)

Figure S4 **Maps of the quadrat trait and soil fertility patterns for the BCI plot.** Map a), b), c), d) and e) are the observed leaf area, SLA, seed mass, wood density and maximum height pattern for the BCI plot; and maps f) and g) are the soil PC1 and PC2 values for the BCI plot. The color scale on the right of each map indicates the trait and soil PC1 and PC2 values. The lines are elevation contour lines at 5-m intervals.
(TIF)

Table S1 **Pearson correlation coefficients between five functional traits and 13 soil nutrients for the GTS plot at the species-level (leaf area and seed mass are log_10_transformed).**
(DOCX)

Table S2 **Pearson correlation coefficients between five functional traits and 13 soil nutrients for the BCI plot at the species-level (leaf area and seed mass are log_10_transformed).**
(DOCX)

Table S3 **Phylogenetically independent contrasts (PICs) between five functional traits and 13 soil nutrients for the GTS plot at the species-level.**
(DOCX)

Table S4 **Phylogenetically independent contrasts (PICs) between five functional traits and 13 soil nutrients for the BCI plot at the species-level.**
(DOCX)

Table S5 **Pearson correlation coefficients between five functional traits and 13 soil nutrients for the GTS plot at the quadrat-level.**
(DOCX)

Table S6 **Pearson correlation coefficients between five functional traits and 13 soil nutrients for BCI plot at the quadrat-level (leaf area and seed mass are log_10_transformed).**
(DOCX)
