# Supplementary figures and images for: Covariation in Plant Functional Traits and Soil Fertility within Two Species-Rich Forests
Source: PLoS One. 2012 Apr 3;7(4):e34767. doi: 10.1371/journal.pone.0034767 (PMC3318000; doi:10.1371/journal.pone.0034767)

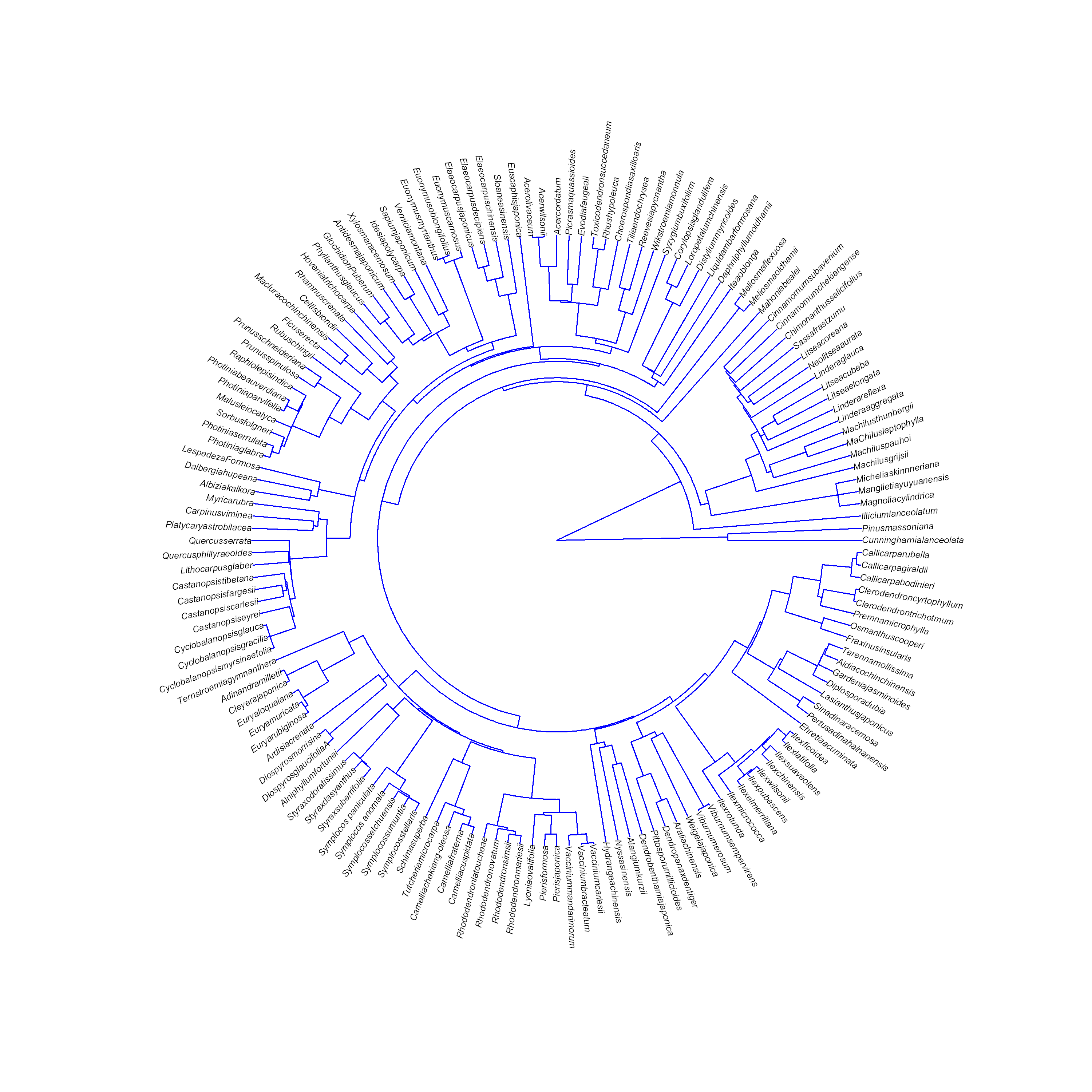

Supplement: Figure S1 — The phylogenetic tree constructed using DNA barcodes of the species in the GTS plot (See details on tree construction in the text). (TIF) [file pone.0034767.s001.tif]

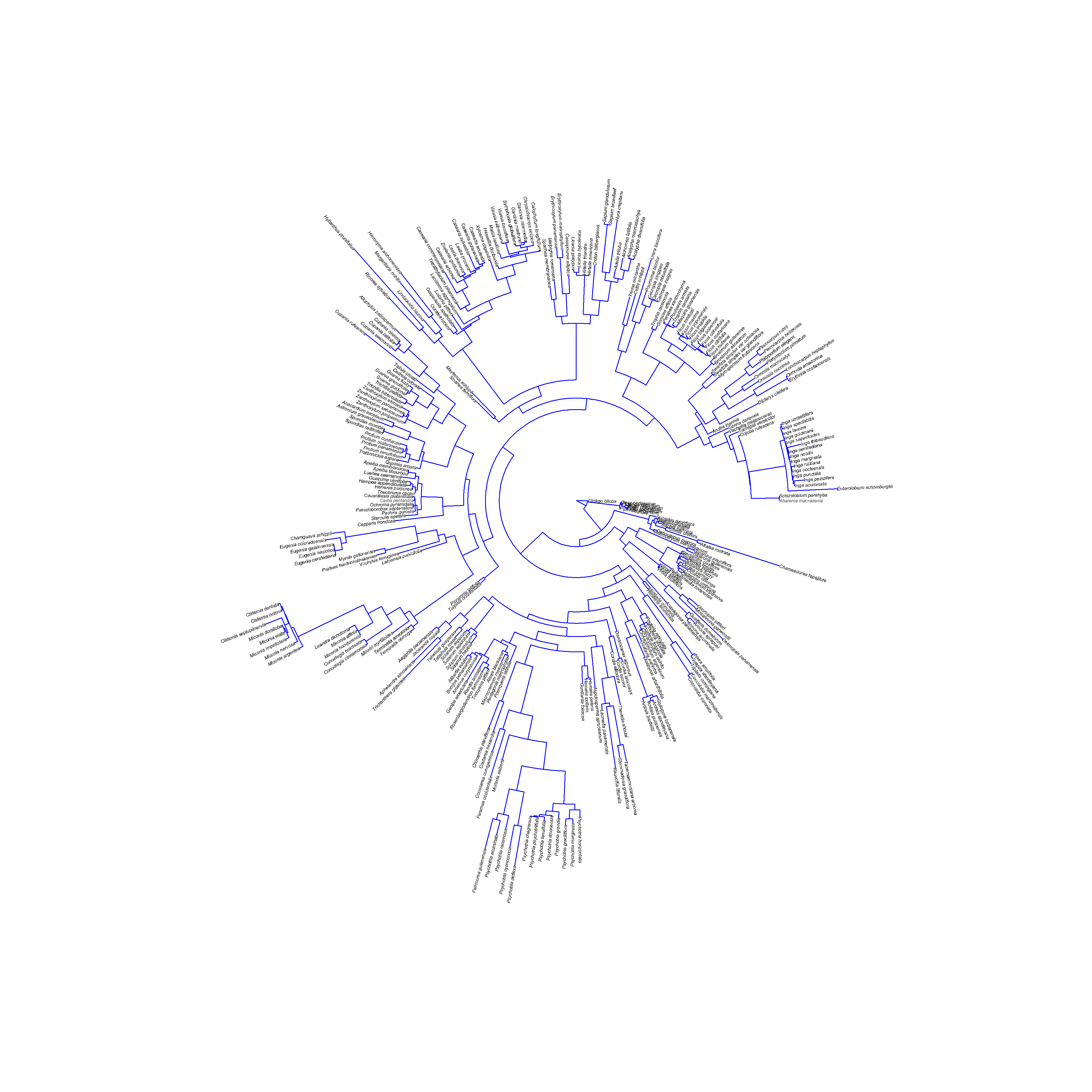

Supplement: Figure S2 — The phylogenetic tree constructed using DNA barcodes of the species in the BCI plot (See details on tree construction in the text). (TIF) [file pone.0034767.s002.tif]

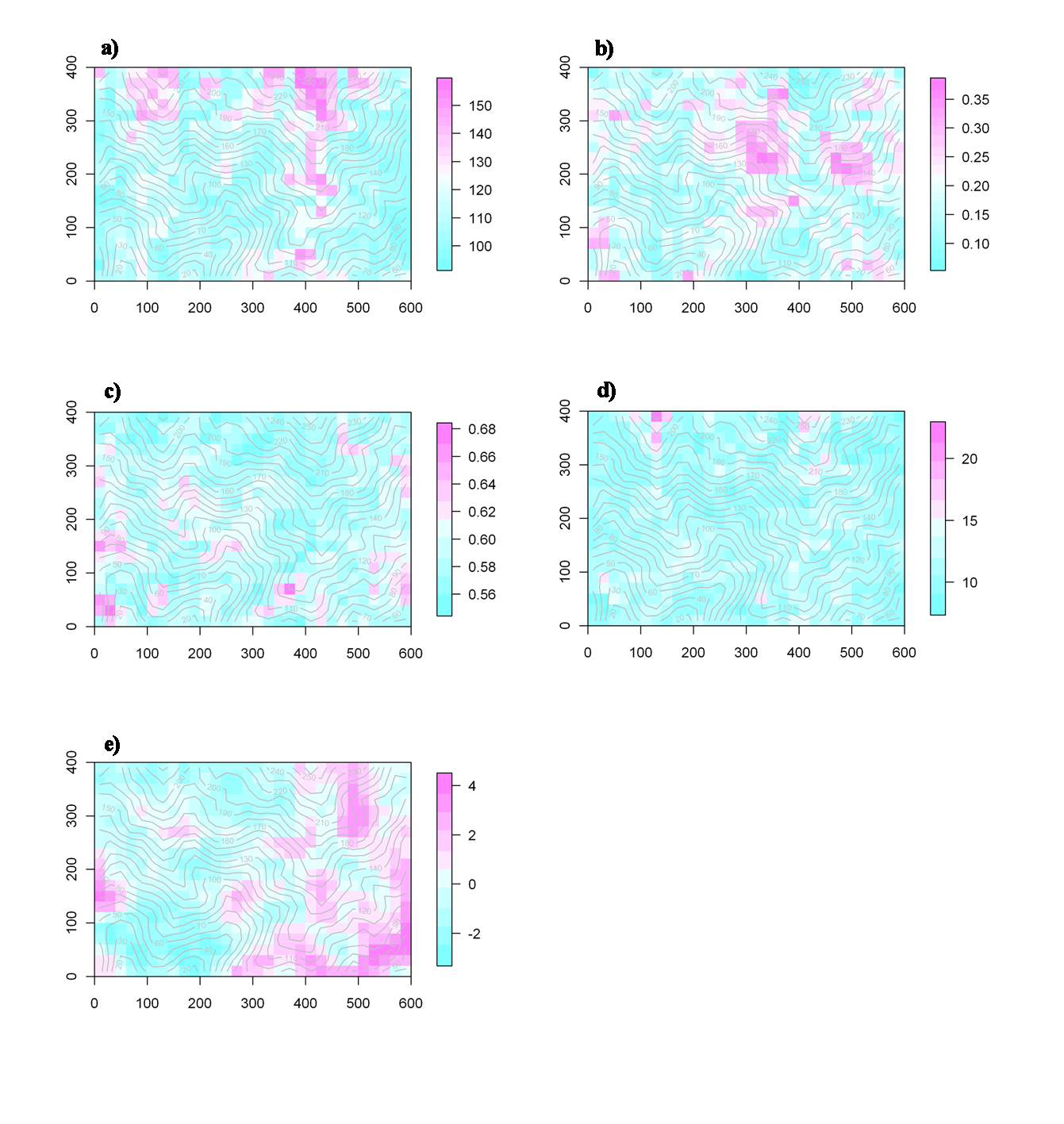

Supplement: Figure S3 — Maps of the quadrat trait and soil fertility patterns for the GTS plot. Map a), b), c) and d) are the observed SLA, seed mass, wood density and maximum height patterns; and map e) is the soil PC2 values for the GTS plot. The color scale on the right of each map indicates the trait and soil PC2 values. The lines are elevation contour lines at 10-m intervals. (TIF) [file pone.0034767.s003.tif]

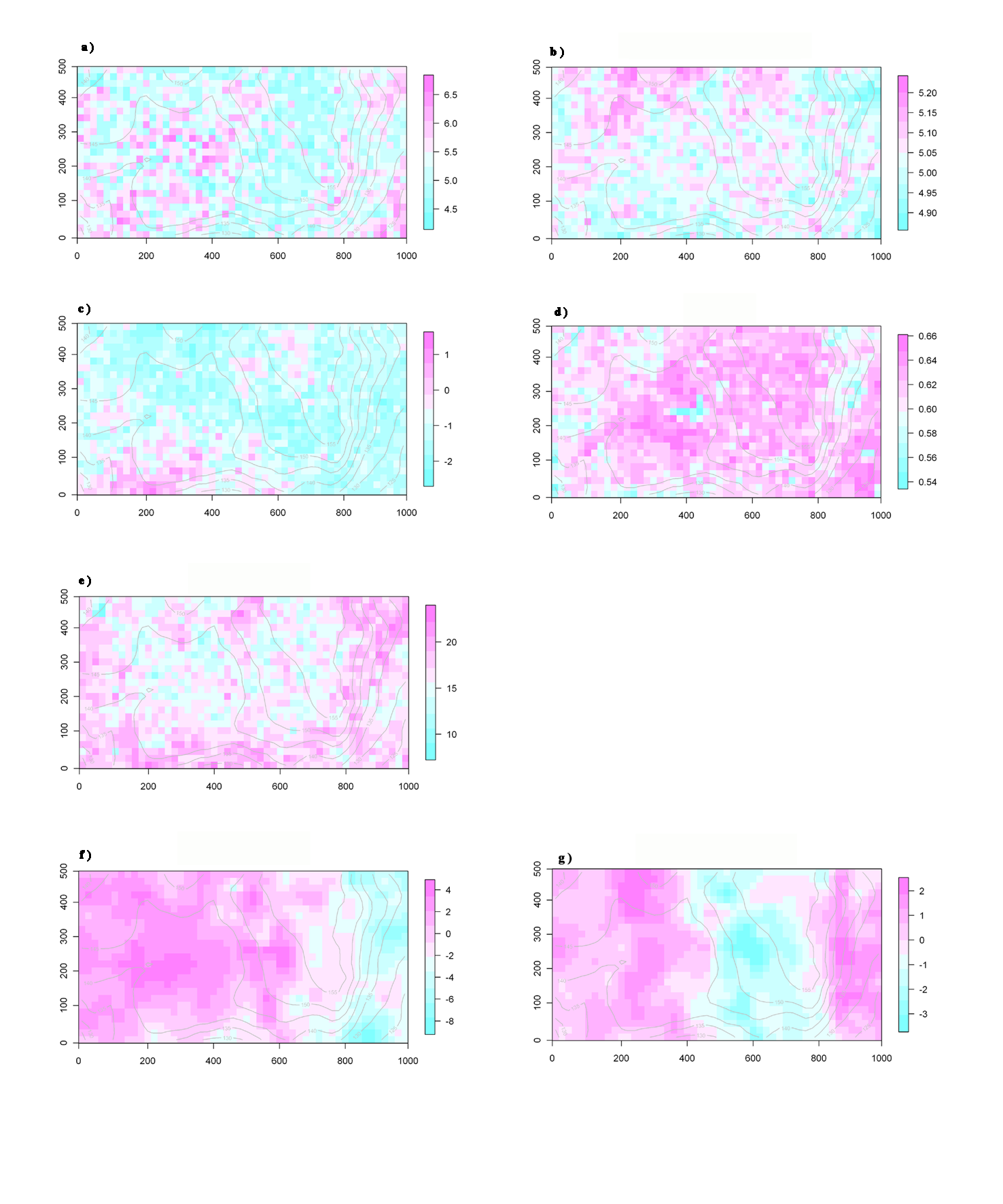

Supplement: Figure S4 — Maps of the quadrat trait and soil fertility patterns for the BCI plot. Map a), b), c), d) and e) are the observed leaf area, SLA, seed mass, wood density and maximum height pattern for the BCI plot; and maps f) and g) are the soil PC1 and PC2 values for the BCI plot. The color scale on the right of each map indicates the trait and soil PC1 and PC2 values. The lines are elevation contour lines at 5-m intervals. (TIF) [file pone.0034767.s004.tif]
