# Supplementary material for: Covariation in Plant Functional Traits and Soil Fertility within Two Species-Rich Forests
Source: PLoS One. 2012 Apr 3;7(4):e34767. doi: 10.1371/journal.pone.0034767 (PMC3318000; doi:10.1371/journal.pone.0034767)
Supplement: Table S1 — Pearson correlation coefficients between five functional traits and 13 soil nutrients for the GTS plot at the species-level (leaf area and seed mass are log10transformed). (DOCX) [file pone.0034767.s005.docx]

Table S1. Pearson correlation coefficients between five functional traits and 13 soil nutrients for the GTS plot at the species-level (leaf area and seed mass are log_10_transformed).

|  |  | Al | B | Ca | Cu | Fe | K | Mg | Mn | P | Zn | N | Nmin | pH |
| --- | --- | --- | --- | --- | --- | --- | --- | --- | --- | --- | --- | --- | --- | --- |
| Leaf area | r | 0.037 | **-0.251** | **0.183** | 0.125 | **-0.348** | **0.218** | **0.205** | **0.240** | **0.205** | 0.078 | **0.224** | **0.140** | **0.180** |
|  | n | 157 | 157 | 157 | 157 | 157 | 157 | 157 | 157 | 157 | 157 | 157 | 157 | 157 |
|  | p | 0.322 | <.001 | 0.011 | 0.059 | <.001 | 0.003 | 0.005 | 0.001 | 0.005 | 0.166 | 0.002 | 0.040 | 0.012 |
| Specific leaf area | r | -0.032 | **-0.133** | -0.014 | 0.037 | **-0.244** | **-0.190** | -0.039 | 0.048 | -0.072 | -0.114 | -0.044 | -0.034 | **0.197** |
|  | n | 157 | 157 | 157 | 157 | 157 | 157 | 157 | 157 | 157 | 157 | 157 | 157 | 157 |
|  | p | 0.345 | 0.048 | 0.431 | 0.323 | 0.001 | 0.009 | 0.314 | 0.275 | 0.185 | 0.078 | 0.292 | 0.336 | 0.007 |
| Seed mass | r | 0.018 | 0.103 | 0.037 | **0.158** | 0.060 | 0.120 | 0.061 | 0.112 | 0.015 | 0.070 | 0.138 | 0.098 | 0.014 |
|  | n | 141 | 141 | 141 | 141 | 141 | 141 | 141 | 141 | 141 | 141 | 141 | 141 | 141 |
|  | p | 0.416 | 0.112 | 0.332 | 0.031 | 0.240 | 0.078 | 0.236 | 0.093 | 0.430 | 0.205 | 0.051 | 0.124 | 0.435 |
| Wood density | r | -0.040 | **0.266** | -0.057 | 0.029 | **0.297** | -0.083 | -0.050 | **-0.145** | -0.024 | 0.112 | -0.050 | -0.023 | **-0.151** |
|  | n | 157 | 157 | 157 | 157 | 157 | 157 | 157 | 157 | 157 | 157 | 157 | 157 | 157 |
|  | p | 0.310 | <.001 | 0.239 | 0.359 | <.001 | 0.151 | 0.267 | 0.035 | 0.383 | 0.081 | 0.267 | 0.388 | 0.030 |
| Maximum height | r | -0.050 | 0.010 | 0.035 | 0.039 | -0.001 | 0.126 | 0.039 | 0.059 | 0.049 | -0.039 | 0.019 | 0.008 | -0.039 |
|  | n | 157 | 157 | 157 | 157 | 157 | 157 | 157 | 157 | 157 | 157 | 157 | 157 | 157 |
|  | p | 0.267 | 0.451 | 0.332 | 0.314 | 0.495 | 0.058 | 0.314 | 0.232 | 0.271 | 0.314 | 0.407 | 0.460 | 0.314 |

* Significant correlations are in boldface type (p-value < 0.05).
