# Supplementary material for: Covariation in Plant Functional Traits and Soil Fertility within Two Species-Rich Forests
Source: PLoS One. 2012 Apr 3;7(4):e34767. doi: 10.1371/journal.pone.0034767 (PMC3318000; doi:10.1371/journal.pone.0034767)
Supplement: Table S2 — Pearson correlation coefficients between five functional traits and 13 soil nutrients for the BCI plot at the species-level (leaf area and seed mass are log10transformed). (DOCX) [file pone.0034767.s006.docx]

Table S2. Pearson correlation coefficients between five functional traits and 13 soil nutrients for the BCI plot at the species-level (leaf area and seed mass are log_10_transformed).

|  |  | Al | B | Ca | Cu | Fe | K | Mg | Mn | P | Zn | N | Nmin | pH |
| --- | --- | --- | --- | --- | --- | --- | --- | --- | --- | --- | --- | --- | --- | --- |
| Leaf area | r | -0.080 | 0.092 | 0.055 | **0.103** | -0.002 | 0.062 | 0.029 | **0.144** | -0.087 | 0.016 | 0.097 | -0.022 | **0.122** |
|  | n | 267 | 267 | 267 | 267 | 267 | 267 | 267 | 267 | 267 | 267 | 267 | 267 | 267 |
|  | p | 0.096 | 0.067 | 0.185 | 0.047 | 0.487 | 0.156 | 0.319 | 0.009 | 0.078 | 0.397 | 0.057 | 0.360 | 0.023 |
| Specific leaf area | r | -0.039 | 0.044 | 0.035 | -0.024 | 0.010 | 0.029 | 0.055 | -0.063 | **0.119** | 0.076 | -0.059 | 0.080 | 0.022 |
|  | n | 268 | 268 | 268 | 268 | 268 | 268 | 268 | 268 | 268 | 268 | 268 | 268 | 268 |
|  | p | 0.263 | 0.237 | 0.284 | 0.348 | 0.435 | 0.318 | 0.185 | 0.152 | 0.026 | 0.108 | 0.168 | 0.096 | 0.360 |
| Seed mass | r | 0.037 | 0.041 | -0.037 | -0.007 | -0.022 | -0.018 | -0.047 | 0.064 | 0.053 | -0.032 | 0.101 | -0.084 | 0.048 |
|  | n | 159 | 159 | 159 | 159 | 159 | 159 | 159 | 159 | 159 | 159 | 159 | 159 | 159 |
|  | p | 0.322 | 0.304 | 0.322 | 0.465 | 0.392 | 0.411 | 0.278 | 0.211 | 0.254 | 0.344 | 0.103 | 0.146 | 0.274 |
| Wood density | r | 0.014 | 0.026 | 0.048 | -0.023 | 0.080 | 0.007 | 0.091 | 0.002 | **0.111** | 0.083 | 0.080 | 0.079 | 0.028 |
|  | n | 244 | 244 | 244 | 244 | 244 | 244 | 244 | 244 | 244 | 244 | 244 | 244 | 244 |
|  | p | 0.414 | 0.343 | 0.228 | 0.360 | 0.107 | 0.457 | 0.078 | 0.487 | 0.042 | 0.098 | 0.107 | 0.109 | 0.332 |
| Maximum height | r | -0.016 | 0.015 | -0.024 | 0.011 | -0.050 | -0.013 | -0.031 | 0.083 | -0.048 | -0.028 | **0.138** | -0.042 | 0.086 |
|  | n | 283 | 283 | 283 | 283 | 283 | 283 | 283 | 283 | 283 | 283 | 283 | 283 | 283 |
|  | p | 0.394 | 0.401 | 0.344 | 0.427 | 0.201 | 0.414 | 0.302 | 0.082 | 0.211 | 0.320 | 0.010 | 0.241 | 0.075 |

* Significant correlations are in boldface type (p-value < 0.05).
