# Supplementary material for: Covariation in Plant Functional Traits and Soil Fertility within Two Species-Rich Forests
Source: PLoS One. 2012 Apr 3;7(4):e34767. doi: 10.1371/journal.pone.0034767 (PMC3318000; doi:10.1371/journal.pone.0034767)
Supplement: Table S3 — Phylogenetically independent contrasts (PICs) between five functional traits and 13 soil nutrients for the GTS plot at the species-level. (DOCX) [file pone.0034767.s007.docx]

Table S3. Phylogenetically independent contrasts (PICs) between five functional traits and 13 soil nutrients for the GTS plot at the species-level.

|  |  | Al | B | Ca | Cu | Fe | K | Mg | Mn | P | Zn | N | Nmin | pH |
| --- | --- | --- | --- | --- | --- | --- | --- | --- | --- | --- | --- | --- | --- | --- |
| Leaf area | r | **0.247** | **-0.434** | **0.188** | 0.066 | **-0.434** | **0.299** | **0.201** | **0.248** | **0.233** | **0.239** | **0.267** | **0.292** | 0.133 |
|  | n | 148 | 148 | 148 | 148 | 148 | 148 | 148 | 148 | 148 | 148 | 148 | 148 | 148 |
|  | p | 0.001 | <.001 | 0.011 | 0.213 | <.001 | <.001 | 0.007 | 0.001 | 0.002 | 0.002 | <.001 | <.001 | 0.054 |
| Specific leaf area | r | 0.125 | -0.050 | **-0.270** | **0.299** | **-0.235** | **-0.457** | **-0.412** | **-0.203** | **-0.203** | **-0.343** | **-0.331** | -0.119 | 0.094 |
|  | n | 148 | 148 | 148 | 148 | 148 | 148 | 148 | 148 | 148 | 148 | 148 | 148 | 148 |
|  | p | 0.125 | 0.273 | <.001 | <.001 | 0.002 | <.001 | <.001 | 0.007 | 0.007 | <.001 | <.001 | 0.075 | 0.128 |
| Seed mass | r | -0.033 | -0.018 | -0.006 | -0.029 | 0.014 | 0.050 | 0.009 | 0.017 | 0.050 | -0.008 | 0.002 | 0.003 | 0.025 |
|  | n | 134 | 134 | 134 | 134 | 134 | 134 | 134 | 134 | 134 | 134 | 134 | 134 | 134 |
|  | p | 0.353 | 0.418 | 0.473 | 0.370 | 0.436 | 0.283 | 0.459 | 0.423 | 0.283 | 0.464 | 0.491 | 0.486 | 0.387 |
| Wood density | r | 0.009 | **0.173** | -0.126 | 0.056 | 0.115 | -0.072 | -0.116 | **-0.225** | -0.047 | **-0.143** | **-0.180** | -0.112 | 0.018 |
|  | n | 148 | 148 | 148 | 148 | 148 | 148 | 148 | 148 | 148 | 148 | 148 | 148 | 148 |
|  | p | 0.457 | 0.018 | 0.064 | 0.250 | 0.082 | 0.192 | 0.080 | 0.003 | 0.285 | 0.042 | 0.014 | 0.088 | 0.414 |
| Maximum height | r | 0.037 | 0.095 | 0.002 | -0.043 | **0.152** | 0.135 | 0.035 | 0.063 | **0.184** | 0.030 | 0.016 | 0.101 | **-0.204** |
|  | n | 148 | 148 | 148 | 148 | 148 | 148 | 148 | 148 | 148 | 148 | 148 | 148 | 148 |
|  | p | 0.328 | 0.125 | 0.490 | 0.302 | 0.033 | 0.051 | 0.336 | 0.223 | 0.013 | 0.359 | 0.424 | 0.111 | 0.006 |

* Significant correlations are in boldface type (p-value < 0.05).
