# Supplementary material for: Covariation in Plant Functional Traits and Soil Fertility within Two Species-Rich Forests
Source: PLoS One. 2012 Apr 3;7(4):e34767. doi: 10.1371/journal.pone.0034767 (PMC3318000; doi:10.1371/journal.pone.0034767)
Supplement: Table S4 — Phylogenetically independent contrasts (PICs) between five functional traits and 13 soil nutrients for the BCI plot at the species-level. (DOCX) [file pone.0034767.s008.docx]

Table S4. Phylogenetically independent contrasts (PICs) between five functional traits and 13 soil nutrients for the BCI plot at the species-level.

|  |  | Al | B | Ca | Cu | Fe | K | Mg | Mn | P | Zn | N | Nmin | pH |
| --- | --- | --- | --- | --- | --- | --- | --- | --- | --- | --- | --- | --- | --- | --- |
| Leaf area | r | -0.052 | **0.130** | 0.091 | **0.213** | **0.161** | **0.115** | 0.081 | **0.228** | -0.102 | 0.016 | -0.039 | 0.052 | 0.076 |
|  | n | 261 | 261 | 261 | 261 | 261 | 261 | 261 | 261 | 261 | 261 | 261 | 261 | 261 |
|  | p | 0.201 | 0.018 | 0.071 | <.001 | 0.005 | 0.032 | 0.096 | <.001 | 0.050 | 0.399 | 0.265 | 0.201 | 0.110 |
| Specific leaf area | r | -0.064 | -0.026 | 0.054 | -0.041 | 0.073 | 0.046 | 0.095 | **-0.239** | 0.100 | **0.108** | **-0.159** | 0.083 | **-0.169** |
|  | n | 262 | 262 | 262 | 262 | 262 | 262 | 262 | 262 | 262 | 262 | 262 | 262 | 262 |
|  | p | 0.151 | 0.338 | 0.192 | 0.254 | 0.120 | 0.229 | 0.063 | <.001 | 0.053 | 0.041 | 0.005 | 0.090 | 0.003 |
| Seed mass | r | 0.042 | -0.096 | -0.104 | -0.066 | **-0.151** | -0.131 | **-0.153** | -0.072 | -0.005 | **-0.186** | -0.064 | **-0.208** | -0.101 |
|  | n | 156 | 156 | 156 | 156 | 156 | 156 | 156 | 156 | 156 | 156 | 156 | 156 | 156 |
|  | p | 0.301 | 0.117 | 0.098 | 0.207 | 0.030 | 0.052 | 0.028 | 0.186 | 0.475 | 0.010 | 0.214 | 0.005 | 0.105 |
| Wood density | r | **0.198** | **-0.323** | **-0.332** | **-0.448** | **-0.441** | **-0.392** | **-0.322** | **-0.354** | **0.197** | **-0.284** | **-0.168** | **-0.349** | **-0.348** |
|  | n | 238 | 238 | 238 | 238 | 238 | 238 | 238 | 238 | 238 | 238 | 238 | 238 | 238 |
|  | p | 0.001 | <.001 | <.001 | <.001 | <.001 | <.001 | <.001 | <.001 | 0.001 | <.001 | 0.005 | <.001 | <.001 |
| Maximum height | r | **0.263** | **0.305** | **0.401** | **0.423** | **0.493** | **0.389** | **0.431** | **0.340** | **0.144** | **0.209** | **0.395** | **0.318** | **0.530** |
|  | n | 156 | 156 | 156 | 156 | 156 | 156 | 156 | 156 | 156 | 156 | 156 | 156 | 156 |
|  | p | <.001 | <.001 | <.001 | <.001 | <.001 | <.001 | <.001 | <.001 | 0.037 | 0.004 | <.001 | <.001 | <.001 |

* Significant correlations are in boldface type (p-value < 0.05).
