# Supplementary material for: Covariation in Plant Functional Traits and Soil Fertility within Two Species-Rich Forests
Source: PLoS One. 2012 Apr 3;7(4):e34767. doi: 10.1371/journal.pone.0034767 (PMC3318000; doi:10.1371/journal.pone.0034767)
Supplement: Table S5 — Pearson correlation coefficients between five functional traits and 13 soil nutrients for the GTS plot at the quadrat-level. (DOCX) [file pone.0034767.s009.docx]

Table S5. Pearson correlation coefficients between five functional traits and 13 soil nutrients for the GTS plot at the quadrat-level.

|  |  | Al | B | Ca | Cu | Fe | K | Mg | Mn | P | Zn | N | Nmin | pH |
| --- | --- | --- | --- | --- | --- | --- | --- | --- | --- | --- | --- | --- | --- | --- |
| Leaf area | r | **0.124** | **-0.412** | **0.366** | **0.350** | **-0.540** | **0.207** | **0.376** | **0.320** | **0.206** | **0.251** | **0.333** | **0.199** | **0.382** |
|  | n | 598 | 598 | 598 | 598 | 598 | 598 | 598 | 598 | 598 | 598 | 598 | 598 | 598 |
|  | p | 0.001 | <.001 | <.001 | <.001 | <.001 | <.001 | <.001 | <.001 | <.001 | <.001 | <.001 | <.001 | <.001 |
| Specific leaf area | r | -0.007 | 0.01 | -0.062 | -0.027 | 0.012 | **-0.422** | **-0.193** | **-0.07** | **-0.378** | **-0.156** | **-0.242** | **-0.362** | **0.057** |
|  | n | 598 | 598 | 598 | 598 | 598 | 598 | 598 | 598 | 598 | 598 | 598 | 598 | 598 |
|  | p | 0.432 | 0.404 | 0.065 | 0.255 | 0.385 | <.001 | <.001 | 0.044 | <.001 | <.001 | <.001 | <.001 | 0.082 |
| Seed mass | r | -0.034 | -0.049 | **0.371** | **0.464** | **-0.241** | **0.424** | **0.435** | **0.535** | **0.087** | **0.238** | **0.345** | **0.104** | **0.126** |
|  | n | 598 | 598 | 598 | 598 | 598 | 598 | 598 | 598 | 598 | 598 | 598 | 598 | 598 |
|  | p | 0.203 | 0.116 | <.001 | <.001 | <.001 | <.001 | <.001 | <.001 | 0.017 | <.001 | <.001 | 0.006 | 0.001 |
| Wood density | r | **0.068** | **0.289** | **0.109** | -0.048 | **0.368** | **0.250** | **0.151** | **-0.109** | **0.304** | **0.266** | **0.175** | **0.331** | **-0.119** |
|  | n | 598 | 598 | 598 | 598 | 598 | 598 | 598 | 598 | 598 | 598 | 598 | 598 | 598 |
|  | p | 0.048 | <.001 | 0.004 | 0.121 | <.001 | <.001 | 0.001 | 0.004 | <.001 | <.001 | <.001 | <.001 | 0.002 |
| Maximum height | r | **-0.299** | **-0.083** | **-0.07** | **-0.11** | -0.021 | **-0.238** | **-0.148** | -0.059 | **-0.200** | **-0.187** | **-0.231** | **-0.096** | 0.042 |
|  | n | 598 | 598 | 598 | 598 | 598 | 598 | 598 | 598 | 598 | 598 | 598 | 598 | 598 |
|  | p | <.001 | 0.021 | 0.044 | 0.004 | 0.304 | <.001 | 0.001 | 0.075 | <.001 | <.001 | <.001 | 0.009 | 0.153 |

* Significant correlations are in boldface type (p-value < 0.05).
