# Supplementary material for: Covariation in Plant Functional Traits and Soil Fertility within Two Species-Rich Forests
Source: PLoS One. 2012 Apr 3;7(4):e34767. doi: 10.1371/journal.pone.0034767 (PMC3318000; doi:10.1371/journal.pone.0034767)
Supplement: Table S6 — Pearson correlation coefficients between five functional traits and 13 soil nutrients for BCI plot at the quadrat-level (leaf area and seed mass are log10transformed). (DOCX) [file pone.0034767.s010.docx]

Table S6. Pearson correlation coefficients between five functional traits and 13 soil nutrients for BCI plot at the quadrat-level (leaf area and seed mass are log_10_transformed).

|  |  | Al | B | Ca | Cu | Fe | K | Mg | Mn | P | Zn | N | Nmin | pH |
| --- | --- | --- | --- | --- | --- | --- | --- | --- | --- | --- | --- | --- | --- | --- |
| Leaf area | r | 0.036 | **-0.104** | 0.020 | **-0.075** | **0.134** | -0.046 | **0.088** | -0.073 | **-0.105** | -0.002 | **-0.215** | 0.036 | **-0.228** |
|  | n | 1248 | 1248 | 1248 | 1248 | 1248 | 1248 | 1248 | 1248 | 1248 | 1248 | 1248 | 1248 | 1248 |
|  | p | 0.102 | <.001 | 0.240 | 0.004 | <.001 | 0.052 | <.001 | 0.005 | <.001 | 0.472 | <.001 | 0.102 | <.001 |
| Specific leaf area | r | **0.214** | **-0.228** | **-0.266** | **-0.065** | **-0.235** | **-0.230** | **-0.309** | **-0.078** | **0.043** | **-0.282** | -0.009 | **-0.243** | **-0.132** |
|  | n | 1248 | 1248 | 1248 | 1248 | 1248 | 1248 | 1248 | 1248 | 1248 | 1248 | 1248 | 1248 | 1248 |
|  | p | <.001 | <.001 | <.001 | <.001 | <.001 | <.001 | <.001 | <.001 | <.001 | <.001 | 0.170 | <.001 | <.001 |
| Seed mass | r | **0.057** | 0.026 | **-0.088** | **-0.108** | **-0.102** | **-0.139** | **-0.063** | **0.077** | **-0.056** | **-0.202** | -**0.120** | **-0.250** | -0.042 |
|  | n | 1248 | 1248 | 1248 | 1248 | 1248 | 1248 | 1248 | 1248 | 1248 | 1248 | 1248 | 1248 | 1248 |
|  | p | 0.022 | 0.179 | <.001 | <.001 | <.001 | <.001 | 0.013 | 0.003 | 0.024 | <.001 | <.001 | <.001 | 0.069 |
| Wood density | r | **0.124** | -0.009 | **-0.019** | **-0.183** | **-0.130** | -0.004 | **0.036** | **-0.138** | **0.329** | **0.123** | **0.174** | **0.140** | **0.047** |
|  | n | 1248 | 1248 | 1248 | 1248 | 1248 | 1248 | 1248 | 1248 | 1248 | 1248 | 1248 | 1248 | 1248 |
|  | p | <.001 | 0.167 | 0.022 | <.001 | <.001 | 0.336 | <.001 | <.001 | <.001 | <.001 | <.001 | <.001 | <.001 |
| Maximum height | r | **-0.308** | **0.260** | **0.275** | **0.217** | **0.252** | **0.252** | **0.258** | **0.231** | **-0.324** | **0.192** | **0.023** | **0.126** | **0.157** |
|  | n | 1248 | 1248 | 1248 | 1248 | 1248 | 1248 | 1248 | 1248 | 1248 | 1248 | 1248 | 1248 | 1248 |
|  | p | <.001 | <.001 | <.001 | <.001 | <.001 | <.001 | <.001 | <.001 | <.001 | <.001 | 0.007 | <.001 | <.001 |

* Significant correlations are in boldface type (p-value < 0.05).
